# Case Report: The combination of upadacitinib and adalimumab in the treatment of refractory ankylosing spondylitis

**DOI:** 10.3389/fimmu.2025.1618725

**Published:** 2025-08-27

**Authors:** Jiajia Wu, Weiwei Wang, Xi Cheng, You Song, Xujing Yuan, Rong Du

**Affiliations:** Department of Rheumatology and Immunology, Union Hospital, Tongji Medical College, Huazhong University of Science and Technology, Wuhan, China

**Keywords:** ankylosing spondylitis, upadacitinib, adalimumab, JAK inhibitors, case report

## Abstract

Ankylosing spondylitis (AS) is a chronic inflammatory disease primarily affecting the axial skeleton and peripheral joints, with potential extra-articular involvement. This study documents the first successful application of upadacitinib-adalimumab combination therapy in refractory AS (disease duration >20 years). Following sequential treatment failures with NSAIDs, TNF inhibitors (adalimumab/etanercept), IL-17 inhibitors (secukinumab), and JAK inhibitors (tofacitinib followed by upadacitinib monotherapy) amidst persistently high disease activity (BASDAI >4 or ASDAS-CRP >2.1), this mechanistically-driven dual-target approach leveraging synergistic JAK-TNF pathway inhibition achieved significant clinical improvement within six months: inflammatory markers decreased substantially (C-reactive protein from 64.6 mg/L to 7.59 mg/L; erythrocyte sedimentation rate from 56 mm/h to 5 mm/h), accompanied by marked reductions in disease activity scores (ASDAS-CRP from 3.9 to 1.77; BASDAI from 3.8 to 1.4). Crucially, rigorous surveillance exceeding 12 months detected no severe adverse events including serious infections or major adverse cardiovascular events. This case establishes targeted combination therapy with intensive safety monitoring as a viable option for multi-drug refractory AS, warranting further validation studies.

## 1 Introduction

Ankylosing spondylitis (AS) is a chronic inflammatory disease primarily affecting the axial skeleton and sacroiliac joints, potentially leading to joint fusion and restricted mobility. The prevalence of AS in the general population ranges from 9 to 30 cases per 10,000 people. It is most commonly diagnosed in people in their 30s and is rarely diagnosed after the age of 45 (1). The disease typically begins in the lower back and sacroiliac regions, with inflammatory processes potentially extending to peripheral joints (e.g., hips and knees) as the condition advances. In the later stages of spinal involvement, the classic “bamboo spine” appearance emerges, significantly impacting the patient’s physical and mental health as well as quality of life. Unlike refractory rheumatoid arthritis, refractory AS currently lacks standardized diagnostic criteria. However, it is broadly defined as persistent disease activity with inadequate symptom control (e.g., unremitting pain, persistent stiffness) despite adherence to standard therapies, including NSAIDs and biologic agents. This may be attributed to factors such as individual variability, disease subtypes, drug tolerance, and drug response (2).

The current biological disease-modifying antirheumatic drugs (bDMARDs) for AS primarily include tumor necrosis factor inhibitors (TNFi) (e.g., adalimumab, etanercept), interleukin-17 inhibitors (IL-17i) (e.g., secukinumab), and Janus kinase inhibitors (JAKi) (e.g., tofacitinib, baricitinib, upadacitinib) (3). While monotherapy with a single biologic agent remains the standard approach, combination therapy with adalimumab and a JAK inhibitor may represent a salvage strategy for refractory AS patients who exhibit inadequate responses to multiple sequential biologic therapies. Here, we present a case of a long-standing AS patient who exhibited inadequate disease control with regular NSAIDs and TNFi therapy. Successful disease management was achieved through combination therapy with the upadacitinib and adalimumab. After more than one year of continuous follow-up, no severe infections or other adverse effects were observed.

## 2 Case report

A 39-year-old Chinese Han male initially developed sacroiliac and hip joint pain accompanied by stiffness, restricted mobility, and inability to squat at age 13 (1999), with characteristic morning stiffness alleviated by physical activity. The patient was diagnosed with AS according to the 1984 modified New York criteria (4), he had no family history of AS or related spondyloarthropathies. Unemployed at presentation, his anthropometrics included height 171 cm, weight 59 kg, and BMI 20.2 kg/m². He reported a 15-year smoking history (approximately 2 cigarettes/day) without significant alcohol use or other notable habits. From 2012 through approximately 2020, treatment regimens comprising etanercept combined with oral medications (diclofenac sodium, prednisone, and methotrexate) and traditional Chinese physiotherapy provided moderate symptomatic relief. In 2020, the patient self-discontinued all medications except etanercept due to perceived insufficient therapeutic efficacy.

**Figure 1:**
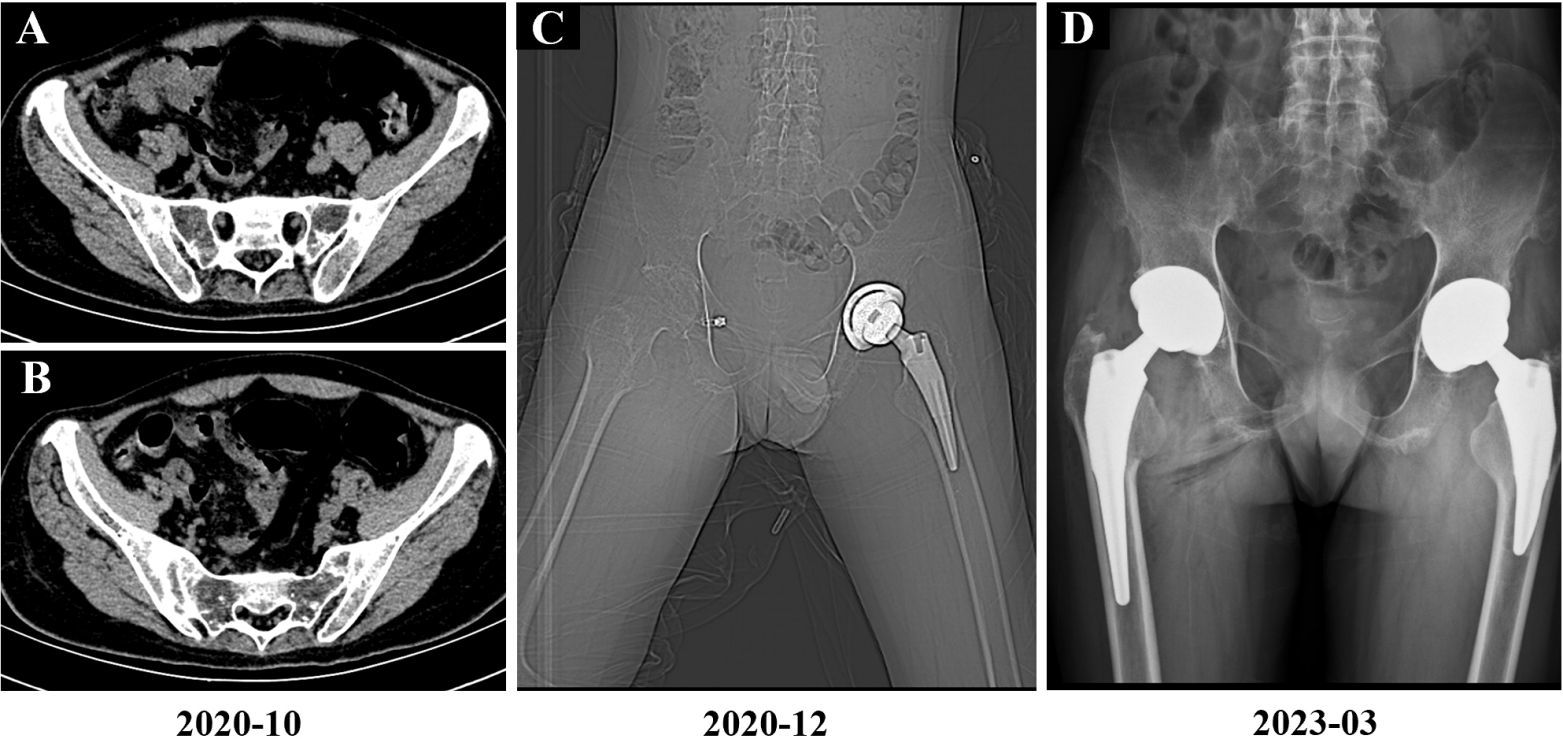
Imaging findings of the patient. **(A, B)** October 2020 sacroiliac joint CT demonstrating bilateral bony ankylosis with complete obliteration of joint spaces. **(C)** December 2020 postoperative imaging following left total hip arthroplasty (CT localizer image). **(D)** March 2023 postoperative imaging following right total hip arthroplasty (anteroposterior radiograph).

**Figure 2:**
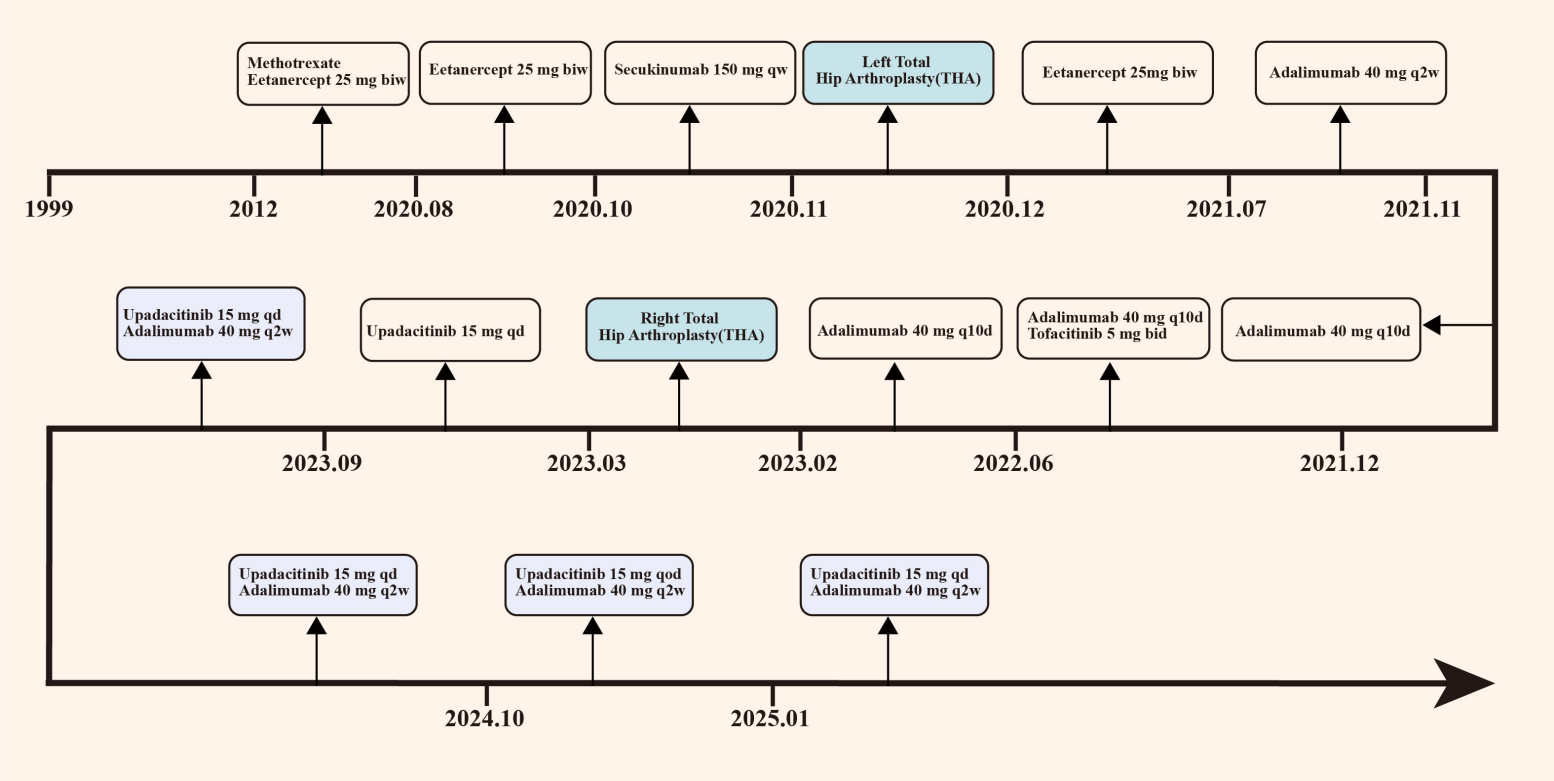
Chronological overview of the patient’s primary treatment protocol. The patient initiated etanercept therapy in 2012, transitioned to secukinumab in October 2020, and switched to adalimumab in July 2021. In December 2021, combination therapy with adalimumab and tofacitinib was commenced. Treatment was further modified to upadacitinib monotherapy in March 2023, followed by combined upadacitinib and adalimumab therapy in September 2023. Notably, the patient underwent left total hip arthroplasty in October 2020 and right total hip arthroplasty in March 2023. biw, twice a week; qw, once a week; qd, once a day; q2w, every 2 weeks; q10d, every 10 days; qod, every other day.

In August 2020, the patient experienced worsening pain in the lumbar and sacroiliac regions, along with swelling and pain in the left knee joint and bilateral wrist joints. Physical examination revealed kyphosis of the thoracic spine, tenderness in both inguinal regions and over the greater trochanters, restricted hip mobility (inability to squat or ambulate without crutches), positive left Thomas test, and incomplete right Patrick’s test. At this time, the Bath Ankylosing Spondylitis Metrology Index (BASMI) score was 6.8. After completing the imaging examination (**Figure 1**), treatment with secukinumab (150 mg administered subcutaneously once weekly) was initiated, but the patient experienced recurrent abdominal pain and diarrhea, which were intolerable, leading to discontinuation of the medication after five doses. Due to persistent hip pain and difficulty walking, the patient underwent left total hip replacement left total hip arthroplasty (THA) in November 2020 (**Figures 1**, **2**), during which glucocorticoids and biologics were withheld perioperatively and for one month postoperatively. Persistent lumbosacral and hip pain led to rheumatology re-evaluation, revealing multijoint involvement (right hip, knees, wrists, elbows, ankles) with bilateral inguinal/greater trochanteric tenderness, restricted hip mobility, positive bilateral Thomas tests, limited Patrick’s tests, impaired occiput-to-wall distance, and reduced chest expansion. Following unsuccessful pain control with regular NSAIDs, etanercept (25 mg administered subcutaneously twice weekly) therapy was reintroduced, yielding partial improvement in joint pain and mobility limitations. However, the patient continued to experience significant restrictions in daily activities and, therefore, he self-administered glucocorticoid therapy. Subsequently, the patient persisted with long-term self-administration of methylprednisolone (4 mg to 24 mg orally once daily), NSAIDs (diclofenac sodium sustained-release tablets 75 mg orally once or twice daily), and etanercept (25 mg administered subcutaneously twice weekly) for six months, yet experienced recurrent symptomatic exacerbations.

In July 2021, due to persistent axial pain, diminished quality of life, and suboptimal response to long-term NSAIDs and etanercept, therapy was transitioned to adalimumab (40 mg administered subcutaneously every two weeks). After five months of treatment, clinical improvement remained unsatisfactory, with elevated inflammatory markers (ESR, CRP) and persistently high ASDAS (ASDAS-CRP 3.82) and BASDAI scores (BASDAI 3.8). Following multidisciplinary consultation and patient-centered discussion, a combined therapeutic regimen of adalimumab (40 mg subcutaneously every 10 days) and tofacitinib (5 mg orally twice daily) was initiated under rigorous monitoring protocol for infection surveillance and adverse event management. Over six months of dual therapy, no severe infections or significant adverse reactions were observed. However, inadequate pain control and failure to sustain low-grade inflammation (BASDAI >4 and ASDAS-CRP >2.1 at discontinuation) led to treatment cessation, primarily due to financial constraints prohibiting continued tofacitinib use. This was followed by disease recurrence, ultimately necessitating right total hip arthroplasty in February 2023 (**Figures 1**, **2**). Given upadacitinib’s enhanced JAK1 selectivity and superior inhibitory potential compared to tofacitinib, coupled with its regulatory approval for AS (5), therapy was transitioned to upadacitinib (15 mg orally once daily) based on active disease status (BASDAI >4 and ASDAS-CRP >2.1) following shared decision-making. Concurrent glucocorticoid tapering was implemented during this therapeutic shift. After six months of treatment, global clinical status improved, though inflammatory markers remained elevated. The patient reported intensified pain perception correlating with ESR elevation (ASDAS-CRP 3.9, ASDAS-ESR 3.47 and BASDAI 3.8), prompting re-evaluation of combination therapy with a JAK inhibitor and biologic agent (adalimumab). Following comprehensive risk-benefit counseling regarding adalimumab-upadacitinib coadministration and obtaining informed consent, the dual-target regimen was initiated (**Figure 2**).

**Figure 3:**
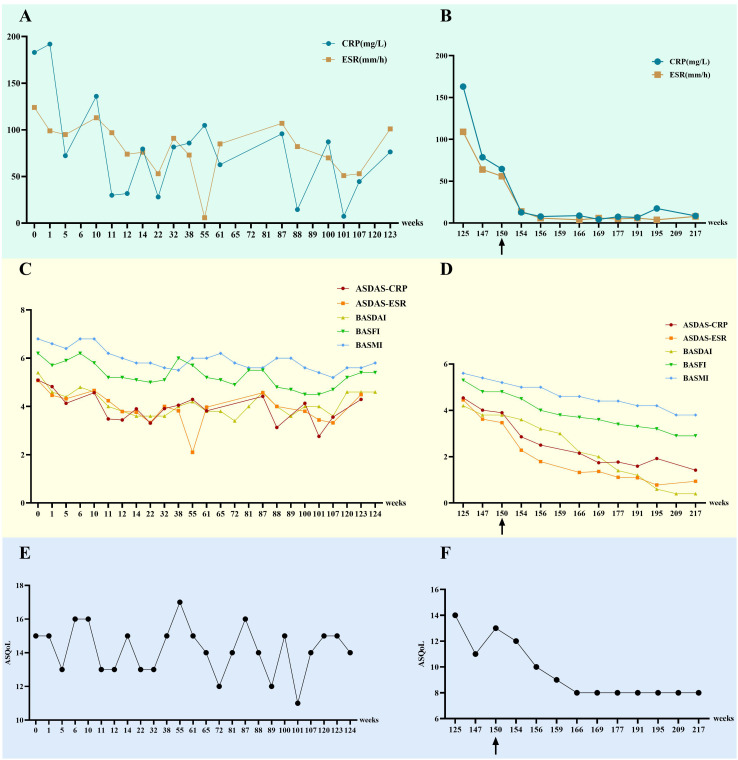
Changes in indicators for this patient before and after the use of upadacitinib. **(A, B)** The changes in CRP and ESR. **(C, D)** The changes in ASDAS-CRP, ASDAS-ESR, BASDAI, BASFI, and BASMI. **(E, F)** The changes in ASQoL.”0 weeks” refers to the patient’s first visit date (October 2020), “125 weeks” refers to the time when the patient first used upadacitinib (March 2023), and “150 weeks” refers to the time when the patient began the combination therapy of adalimumab and upadacitinib. CRP, C-reactive protein (normal range: <8.00 mg/L); ESR, erythrocyte sedimentation rate (normal range: <15 mm/h); ASDAS-CRP, AS Disease Activity Score-CRP; ASDAS-ESR, AS Disease Activity Score-ESR; BASDAI, Bath AS Disease Activity Index; BASFI, Bath AS Functional Index; BASMI, Bath AS Metrology Index; ASQoL, Ankylosing Spondylitis Quality of Life.

Following initiation of combination therapy with upadacitinib and adalimumab, the patient demonstrated marked symptomatic improvement. At approximately 6 months post-treatment initiation, significant reductions in inflammatory markers were observed: CRP decreased from 64.6 mg/L to 7.59 mg/L (normal range: <8.00 mg/L) and ESR declined from 56 mm/h to 5 mm/h (normal range: <15 mm/h), both stabilizing within normal ranges. Concurrently, progressive declines were noted in standardized assessment scores: ASDAS-CRP (3.90 to 1.77), ASDAS-ESR (3.47 to 1.11), BASDAI (3.8 to 1.4), BASFI (4.8 to 3.4), BASMI (5.2 to 4.4), and ASQoL (13 to 8) (**Figure 3**). The patient also gradually tapered off the self-administered glucocorticoids until complete discontinuation. The patient attempted dose reduction of upadacitinib (15 mg orally every other day), however, by January 2025, axial pain exacerbation was observed during outpatient follow-up, prompting re-escalation to the standard dose (15 mg orally once daily). To date, no treatment-related infections or adverse events have been documented, with ongoing close clinical surveillance (**Figure 2**).

## 3 Discussion

To the best of our knowledge, this is the first reported case of refractory AS effectively controlled by a combination of adalimumab and upadacitinib. Previous reports have all involved monotherapy with a single biologic, making our case particularly noteworthy as it highlights the potential efficacy of two biologic agents in managing refractory AS, which is challenging and severely impacts the quality of life.

AS exhibits marked clinical heterogeneity, manifesting in both disease phenotypes and therapeutic responses. This necessitates individualized management—a principle endorsed by international guidelines including the EULAR recommendations (Recommendation 1) (3). Regarding treatment-refractory AS, no consensus definitions akin to those for rheumatoid arthritis (RA) exist, nor are there specific therapeutic recommendations for this subset beyond surgical intervention. In this case, sequential use of TNFi and IL-17i both failed (BASDAI > 4 or ASDAS-CRP > 2.1), underscoring the need for effective alternatives (6).

TNF inhibitors (TNFi) and JAK inhibitors (JAKi) have both demonstrated efficacy in ankylosing spondylitis (AS) through randomized controlled trials (RCTs) and real-world data. Mechanistically, these agents engage distinct inflammatory signaling pathways, with JAKi exhibiting synergistic effects on the suppression of TNF-mediated pathways (7, 8). TNF-α plays a critical role in the pathogenesis of AS, as it can activate the NF-κB and MAPK signaling pathways, promoting the release of pro-inflammatory cytokines (e.g., IL-6, IL-17, and IL-23), which leads to chronic inflammation in the spine and sacroiliac joints. The therapeutic efficacy of TNF-α, IL-17, and IL-23 blockade in clinical practice substantiates the critical involvement of these molecules in disease pathophysiology. Notably, the biological functions of these cytokines depend on JAK molecule activation, which mediates intracellular signaling following receptor engagement. Pharmacological agents capable of inhibiting multiple cytokines implicated in axial spondyloarthritis (axSpA) pathogenesis may demonstrate superior efficacy compared to single-target biologics. Substantial evidence implicates JAK molecules as pivotal contributors to axSpA pathogenesis. Genetic mutation studies have shown that polymorphisms in JAK2, TYK2, and STAT3 are associated with the onset of AS (9, 10). Previous studies have established a definite role for TNF-α in the pathogenesis of axSpA, but the receptors associated with the JAK-STAT pathway remain unidentified. However, IL-12 signaling through the JAK2/TYK2 pathway and IFN-γ signaling through JAK1/JAK2 are crucial for macrophages to produce TNF-α (11). Therefore, blocking JAK2/TYK2 or JAK1/JAK2 can indirectly regulate TNF-α production by inhibiting the production of IL-12 and IFN-γ. Consequently, the simultaneous use of TNFi and JAKi may suppress the inflammatory response through different pathways, potentially resulting in additive or synergistic effects (12).

For AS patients with persistent high disease activity despite NSAIDs treatment, the use of bDMARDs, such as TNFi or IL-17i, is recommended (3, 13). Nevertheless, a substantial proportion of patients exhibit inadequate response to first-line bDMARDs, as exemplified by our case where prolonged etanercept use failed to achieve disease control. In the 2022 recommendations from the Assessment of SpondyloArthritis international Society (ASAS)–European Alliance of Associations for Rheumatology (EULAR), the addition of JAKi as an option for patients with NSAIDs intolerance or inadequate response (IR) was included (3). This guidance further advocates switching to alternative bDMARDs or JAKi for patients with IR to initial biologic therapy (14). JAK inhibitors, categorized as targeted synthetic DMARDs (tsDMARDs), exert therapeutic effects via JAK/STAT pathway modulation and have demonstrated clinical potential in axSpA. Upadacitinib, a next-generation selective JAK1 inhibitor, exhibits enhanced JAK1 selectivity compared to tofacitinib, theoretically optimizing its risk-benefit profile (15, 16). Currently, no randomized controlled trials (RCTs) have directly compared different JAKi in patients with AS. Network meta-analyses indirectly evaluating the efficacy of upadacitinib against other JAKi or biologic agents have generated discordant results (17–19). One meta-analysis demonstrated that tofacitinib was most likely to be the optimal JAKi for achieving ASAS40 response, with upadacitinib ranking second (18). Conversely, another study reported that upadacitinib achieved superior ASAS40 response rates compared to alternative therapeutic agents, irrespective of prior biological exposure (19). Furthermore, upadacitinib was associated with the highest proportion of patients attaining ASDAS-defined low disease activity (LDA), regardless of previous TNFi treatment (17). Several clinical trials have validated its efficacy and safety (20). As evidenced by the SELECT-AXIS 1 trial—a multicenter, randomized, double-blind, placebo-controlled phase 2/3 study—upadacitinib 15 mg demonstrated efficacy and tolerability in patients with active AS who had inadequate response or contraindication to non-steroidal anti-inflammatory drugs, supporting its further investigation for axial spondyloarthritis (21). The open-label extension of the SELECT-AXIS 2 study further confirmed sustained efficacy through 104 weeks in biologic-refractory AS patients, including low rates of radiographic progression, with maintained tolerability and no newly identified safety signals (2). Collectively, these findings establish upadacitinib as a treatment option for biologic-resistant AS. In this case, after insufficient response to regular NSAIDs and bDMARDs therapy, tofacitinib was added, but the results were still unsatisfactory. Subsequent six-month upadacitinib monotherapy also proved insufficient. Based on the proposed pathogenesis of AS and previous studies reporting favorable signals with bDMARDs combination regimens in autoimmune diseases (22–25), combined with the consideration that AS patients are generally younger than those with RA and exhibit lower frequency and reduced severity of adverse events, we opted for off-label concomitant use of both adalimumab and upadacitinib after comprehensive benefit-risk assessment and detailed discussion with the patient.

Regarding the adverse reactions and risks associated with the use of TNFi and JAKi, Ytterberg et al. demonstrated elevated risks of major adverse cardiovascular events (MACE) and malignancies with tofacitinib versus TNFi in high cardiovascular-risk populations, alongside higher incidence rates of specific adverse events in the tofacitinib cohort (26). Upadacitinib demonstrated efficacy and an acceptable safety profile for AS patients in the SELECT-AXIS 1 and 2 trials, including patients with inadequate response (IR) to bDMARDs for up to 1 year in the SELECT-AXIS 2 trial (2). Concomitant TNFi and JAKi therapy carries potential additive risks, including increased susceptibility to infections, thromboembolic complications (e.g., deep vein thrombosis, pulmonary embolism), malignancy development, hepatotoxicity potentiation, and cardiovascular/cerebrovascular adverse events. Mechanistically, TNF-α exerts pro-inflammatory effects primarily through the MAPK and NF-κB pathways, critically mediating macrophage and granulocyte recruitment/activation, effector T-cell stimulation, and pro-inflammatory cytokine release. Concurrently, it demonstrates anti-tumor activity by promoting apoptosis (7). The JAK-STAT pathway plays pivotal roles in inflammation, cellular differentiation/proliferation, and hematopoiesis, with the JAK1 subtype driving inflammation via activation by IFN, IL-6, and other cytokines, thereby regulating innate, cellular, and humoral immunity (8). Previous studies indicate that TNFi and JAKi therapy for AS/RA carries risks of infections, viral reactivation, malignancies, cardiovascular events, and thrombosis. These safety concerns warrant heightened vigilance during combination therapy (27, 28). Consistent with this, similar studies report significantly higher adverse event rates with combination regimens versus monotherapy (28). In our case, after multiple therapeutic failures with NSAIDs, TNFi (adalimumab/etanercept), and secukinumab monotherapies, multidisciplinary consultation and detailed patient counseling preceded initiation of upadacitinib-adalimumab combination therapy. Concurrently, intensified monitoring protocols were implemented, including shortened follow-up intervals and rigorous pre-treatment screening for infections and thrombotic risks. The regimen achieved clinically meaningful disease modification, with no severe adverse events (including serious infections or MACE)

In current AS management with TNFi, IL-17i, or JAKi, the proportion of patients achieving low disease activity (LDA) at 3 months ranges from 40-50% (21, 29). This response rate further declines with increasing lines of therapy. While some patients require switching to alternative bDMARDs/tsDMARDs after suboptimal responses, others experience treatment failure despite multiple agent changes. Therapeutically, AS demands both long-term structural progression delay and prompt short-term pain relief (3). Treatment failure thus imposes substantial burdens on both objectives, creating an urgent unmet need for effective strategies in this multidrug-refractory population. Although TNFi and JAKi monotherapies demonstrate established efficacy, combination therapy—following thorough benefit-risk assessment—represents a novel approach for these patients. Regarding safety, while some studies report increased risks with combination regimens (22–25), others document contradictory findings in case reports. These divergent outcomes may relate to specific drug combinations. In our case, the manageable safety profile provides valuable clinical reference for such therapeutic decisions.

In summary, we present a case of a long-standing AS patient who achieved sustained clinical improvement through combination therapy with upadacitinib and adalimumab, enabling progressive tapering and discontinuation of glucocorticoids and NSAIDs. This case provides novel therapeutic insights for refractory AS management. Despite its innovativeness, current ASAS/EULAR guidelines do not endorse concomitant JAKi-TNFi therapy, consequently imposing limitations on broader clinical application. Future large-scale, multicenter randomized controlled trials are warranted to validate the efficacy and safety profile of such combination regimens. Furthermore, continued exploration of emerging targeted therapies and innovative treatment strategies remains imperative to expand therapeutic options for this challenging patient population.

## 4 Patient’s perspective

After enduring refractory ankylosing spondylitis for over two decades with severe limitations in daily functioning, the combination therapy of upadacitinib and adalimumab has provided substantial clinical improvement. Although low back pain persists during vigorous physical activity, I now achieve complete independence in all activities of daily living. This regimen restored my autonomy when other therapeutic strategies had failed.

## Data Availability

The original contributions presented in the study are included in the article/supplementary material. Further inquiries can be directed to the corresponding author.

## References

[1] He W Yang H Yang X Huang J Wu Z. Global research trends in biological therapy for ankylosing spondylitis: A comprehensive visualization and bibliometric study (2004-2023). Hum Vaccin Immunother. (2025) 21:2445900. doi: 10.1080/21645515.2024.2445900, PMID: 39813123 PMC11740677

[2] Baraliakos X van der Heijde D Sieper J Inman RD Kameda H Maksymowych WP. Efficacy and safety of upadacitinib in patients with active ankylosing spondylitis refractory to biologic therapy: 2-year clinical and radiographic results from the open-label extension of the select-axis 2 study. Arthritis Res Ther. (2024) 26:197. doi: 10.1186/s13075-024-03412-8, PMID: 39533349 PMC11556075

[3] Ramiro S Nikiphorou E Sepriano A Ortolan A Webers C Baraliakos X. Asas-eular recommendations for the management of axial spondyloarthritis: 2022 update. Ann Rheum Dis. (2023) 82:19–34. doi: 10.1136/ard-2022-223296, PMID: 36270658

[4] van der Linden S Valkenburg HA Cats A. Evaluation of diagnostic criteria for ankylosing spondylitis. A proposal for modification of the new york criteria. Arthritis Rheum. (1984) 27:361–8. doi: 10.1002/art.1780270401, PMID: 6231933

[5] Braun J Kiltz U Baraliakos X. Management of axial spondyloarthritis - insights into upadacitinib. Drug Des Devel Ther. (2022) 16:3609–20. doi: 10.2147/DDDT.S330413, PMID: 36268520 PMC9578786

[6] Smolen JS Landewe RBM Bergstra SA Kerschbaumer A Sepriano A Aletaha D. Eular recommendations for the management of rheumatoid arthritis with synthetic and biological disease-modifying antirheumatic drugs: 2022 update. Ann Rheum Dis. (2023) 82:3–18. doi: 10.1136/ard-2022-223356, PMID: 36357155

[7] Siegmund D Wajant H. Tnf and tnf receptors as therapeutic targets for rheumatic diseases and beyond. Nat Rev Rheumatol. (2023) 19:576–91. doi: 10.1038/s41584-023-01002-7, PMID: 37542139

[8] McInnes IB Szekanecz Z McGonagle D Maksymowych WP Pfeil A Lippe R. A review of jak-stat signalling in the pathogenesis of spondyloarthritis and the role of jak inhibition. Rheumatol (Oxford). (2022) 61:1783–94. doi: 10.1093/rheumatology/keab740, PMID: 34668515 PMC9071532

[9] Paroli M Caccavale R Paroli MP Spadea L Accapezzato D. Janus kinase inhibitors: A new tool for the treatment of axial spondyloarthritis. Int J Mol Sci. (2023) 24:1027. doi: 10.3390/ijms24021027, PMID: 36674537 PMC9866163

[10] Dendrou CA Cortes A Shipman L Evans HG Attfield KE Jostins L. Resolving tyk2 locus genotype-to-phenotype differences in autoimmunity. Sci Transl Med. (2016) 8:363ra149. doi: 10.1126/scitranslmed.aag1974, PMID: 27807284 PMC5737835

[11] Veale DJ McGonagle D McInnes IB Krueger JG Ritchlin CT Elewaut D. The rationale for janus kinase inhibitors for the treatment of spondyloarthritis. Rheumatol (Oxford). (2019) 58:197–205. doi: 10.1093/rheumatology/key070, PMID: 29618084 PMC6343466

[12] Toussirot E. The use of janus kinase inhibitors in axial spondyloarthritis: current insights. Pharm (Basel). (2022) 15:270. doi: 10.3390/ph15030270, PMID: 35337068 PMC8951918

[13] Ward MM Deodhar A Gensler LS Dubreuil M Yu D Khan MA. 2019 Update of the american college of rheumatology/Spondylitis association of america/Spondyloarthritis research and treatment network recommendations for the treatment of ankylosing spondylitis and nonradiographic axial spondyloarthritis. Arthritis Care Res (Hoboken). (2019) 71:1285–99. doi: 10.1002/acr.24025, PMID: 31436026 PMC6764857

[14] Baraliakos X van der Heijde D Sieper J Inman RD Kameda H Li Y. Efficacy and safety of upadacitinib in patients with ankylosing spondylitis refractory to biologic therapy: 1-year results from the open-label extension of a phase iii study. Arthritis Res Ther. (2023) 25:172. doi: 10.1186/s13075-023-03128-1, PMID: 37723577 PMC10506267

[15] Parmentier JM Voss J Graff C Schwartz A Argiriadi M Friedman M. *In vitro* and *in vivo* characterization of the jak1 selectivity of upadacitinib (Abt-494). BMC Rheumatol. (2018) 2:23. doi: 10.1186/s41927-018-0031-x, PMID: 30886973 PMC6390583

[16] Mohamed MF Beck D Camp HS Othman AA. Preferential inhibition of jak1 relative to jak3 by upadacitinib: exposure-response analyses of ex vivo data from 2 phase 1 clinical trials and comparison to tofacitinib. J Clin Pharmacol. (2020) 60:188–97. doi: 10.1002/jcph.1513, PMID: 31448433 PMC6973126

[17] Baraliakos X Saffore CD Collins EB Parikh B Ye X Walsh JA. Comparative efficacy of advanced therapies in the treatment of radiographic axial spondyloarthritis or ankylosing spondylitis as evaluated by the asdas low disease activity criteria. Rheumatol Ther. (2024) 11:989–99. doi: 10.1007/s40744-024-00685-y, PMID: 38858318 PMC11264655

[18] Lee YH. Comparative efficacy and safety of janus kinase inhibitors and secukinumab in patients with active ankylosing spondylitis: A systematic review and meta-analysis. Pharmacology. (2022) 107:537–44. doi: 10.1159/000525627, PMID: 35817017 PMC9811419

[19] Walsh JA Saffore CD Collins EB Ostor A. Clinical and economic benefit of advanced therapies for the treatment of active ankylosing spondylitis. Rheumatol Ther. (2023) 10:1385–98. doi: 10.1007/s40744-023-00586-6, PMID: 37568031 PMC10468449

[20] Tang H Liu X Zhao J Tang Z Zheng Z Bai W. Upadacitinib for axial spondyloarthritis: A meta-analysis of efficacy and safety. Clin Rheumatol. (2024) 43:2391–402. doi: 10.1007/s10067-024-07027-x, PMID: 38874670

[21] van der Heijde D Song IH Pangan AL Deodhar A van den Bosch F Maksymowych WP. Efficacy and safety of upadacitinib in patients with active ankylosing spondylitis (Select-axis 1): A multicentre, randomised, double-blind, placebo-controlled, phase 2/3 trial. Lancet. (2019) 394:2108–17. doi: 10.1016/S0140-6736(19)32534-6, PMID: 31732180

[22] Barroso NS Miller EZ Furst DE. A case series on patients on tofacitinib in combination with a biologic. J Clin Rheumatol. (2018) 24:349–51. doi: 10.1097/RHU.0000000000000663, PMID: 29280829

[23] Shurey M Yip A Ziouzina O Chan J Dutz JP. Combination therapy with tofacitinib and il-12/23, il-23, or il-17a inhibition for the treatment of refractory psoriatic arthritis: A case series. J Clin Rheumatol. (2022) 28:e626–e8. doi: 10.1097/RHU.0000000000001767, PMID: 34176886

[24] Kim Y Yi H Jung H Rim YA Park N Kim J. A dual target-directed agent against interleukin-6 receptor and tumor necrosis factor alpha ameliorates experimental arthritis. Sci Rep. (2016) 6:20150. doi: 10.1038/srep20150, PMID: 26841833 PMC4740770

[25] Leal S Ivorra-Cortes J de la Rubia-Navarro M Pavez-Perales C Riesco-Barcena C Roman-Ivorra JA. Jak inhibitors in combination with anti-tnf drugs on difficult-to-treat chronic polyarthritis: A case series. Clin Exp Rheumatol. (2024) 42:1135–6. doi: 10.55563/clinexprheumatol/4ao14k, PMID: 38019156

[26] Ytterberg SR Bhatt DL Mikuls TR Koch GG Fleischmann R Rivas JL. Cardiovascular and cancer risk with tofacitinib in rheumatoid arthritis. N Engl J Med. (2022) 386:316–26. doi: 10.1056/NEJMoa2109927, PMID: 35081280

[27] Burmester GR Cohen SB Winthrop KL Nash P Irvine AD Deodhar A. Safety Profile of Upadacitinib over 15–000 Patient-Years across Rheumatoid Arthritis, Psoriatic Arthritis, Ankylosing Spondylitis and Atopic Dermatitis. RMD Open. (2023) 9:e002735. doi: 10.1136/rmdopen-2022-002735, PMID: 36754548 PMC9923346

[28] Boleto G Kanagaratnam L Drame M Salmon JH. Safety of combination therapy with two bdmards in patients with rheumatoid arthritis: A systematic review and meta-analysis. Semin Arthritis Rheum. (2019) 49:35–42. doi: 10.1016/j.semarthrit.2018.12.003, PMID: 30638975

[29] van der Heijde D Baraliakos X Sieper J Deodhar A Inman RD Kameda H. Efficacy and safety of upadacitinib for active ankylosing spondylitis refractory to biological therapy: A double-blind, randomised, placebo-controlled phase 3 trial. Ann Rheum Dis. (2022) 81:1515–23. doi: 10.1136/ard-2022-222608, PMID: 35788492 PMC9606523

